# Supplementary material for: Bevacizumab-induced proteinuria and its association with antihypertensive drugs: A retrospective cohort study using a Japanese administrative database
Source: PLoS One. 2023 Aug 10;18(8):e0289950. doi: 10.1371/journal.pone.0289950 (PMC10414654; doi:10.1371/journal.pone.0289950)
Supplement: S1 Checklist — (DOCX) [file pone.0289950.s001.docx]

STROBE Statement—checklist of items that should be included in reports of observational studies

|  | Item No. | Recommendation | Page  No. | Relevant text from manuscript |
| --- | --- | --- | --- | --- |
| **Title and abstract** | 1 | (*a*) Indicate the study’s design with a commonly used term in the title or the abstract | 1 | Bevacizumab-induced proteinuria and its association with antihypertensive drugs: a retrospective cohort study using a Japanese administrative database |
|  |  | (*b*) Provide in the abstract an informative and balanced summary of what was done and what was found | 2, 3 | Proteinuria is a major side-effect of the anti-tumor drug bevacizumab, although its incidence and risk factors in the real world are still unclear. Although renin-angiotensin-aldosterone system inhibitors are used clinically to prevent proteinuria, their efficacy remains unclear. The aim of the present study was to reveal the incidence and risk factors of bevacizumab-induced proteinuria and examine the effectiveness of antihypertensive drugs in preventing proteinuria. We conducted a retrospective cohort study using the National Hospital Organization Clinical Data Archives and Medical Information Analysis Databank. Hospitalized patients who received bevacizumab between January 1, 2016 and June 30, 2019 were included. The study outcome was proteinuria within 12 months of bevacizumab administration. Patient characteristics, laboratory tests, and medications were compared between patients with and without proteinuria using multivariable logistic regression analysis. Among the 2,458 patients, 27% developed proteinuria after bevacizumab administration. Nursing dependence (odds ratio [OR], 2.40; 95% confidence interval [CI], 1.89–3.05; P<0.001) and systolic blood pressure ≥140 mmHg (OR, 1.46; 95% CI, 1.18–1.80; P<0.001) were identified as risk factors. Patients with an estimated glomerular filtration rate (eGFR) of 60–89, 45–59, and <45 mL/min/1.73 m^2^ had 32.0%, 82.4%, and 70.8% higher odds of proteinuria, respectively, than those with an eGFR ≥90 mL/min/1.73 m^2^. No significant relationship was observed between antihypertensive drugs and the occurrence of proteinuria. More patients may suffer from proteinuria after bevacizumab administration than previously reported. Nursing dependence and systolic blood pressure are predictive risk factors for bevacizumab-induced proteinuria. Patients at-risk of proteinuria should be closely monitored. |
| Introduction | | | |  |
| Background/rationale | 2 | Explain the scientific background and rationale for the investigation being reported | 3, 4 | Bevacizumab, a vascular endothelial growth factor inhibitor, is widely administered for the treatment of colorectal, non-small cell lung, breast, and ovarian cancers [1-4]. Hypertension and proteinuria are the major side effects of bevacizumab. The incidence of proteinuria is 10.4% according to the package insert, although a meta-analysis of 16 randomized controlled trials reported an incidence of 13.3% [5]. However, the actual incidence and onset time of bevacizumab-induced proteinuria remain unclear.  Antihypertensive drugs have been empirically administered to prevent and treat bevacizumab-induced proteinuria [6]. Renin-angiotensin-aldosterone system (RAA) inhibitors (including angiotensin receptor blockers [ARBs] and angiotensin-converting enzyme inhibitors [ACEIs]), which increase nitric oxide levels, have been reported to prevent and treat bevacizumab-induced proteinuria [7]. However, bevacizumab indirectly inhibits endothelial nitric oxide synthase, lowering nitric oxide levels [8,9]. Thus, RAA inhibitors may antagonize bevacizumab activity and lower its efficacy [10]. Moreover, bevacizumab-induced proteinuria has been studied in limited populations [6,7,10], and a real-world investigation on the appropriate antihypertensive drug for preventing/treating bevacizumab-induced proteinuria is required.  Real-world evidence is a valuable tool for assessing drug effectiveness and safety, especially among patients who are often excluded from randomized controlled trials [11]. Such evidence using daily healthcare service provisions, laboratory data, and patient medical records reflects the actual effect and safety of the drug in patients [11]. |
| Objectives | 3 | State specific objectives, including any prespecified hypotheses | 4 | The present study investigated the risk factors of bevacizumab-induced proteinuria and examined the effectiveness of antihypertensive drugs in preventing proteinuria using a Japanese nationwide administrative database. |
| Methods | | | |  |
| Study design | 4 | Present key elements of study design early in the paper | 5 | The retrospective cohort study |
| Setting | 5 | Describe the setting, locations, and relevant dates, including periods of recruitment, exposure, follow-up, and data collection | 5 | This retrospective cohort study used data from the National Hospital Organization (NHO) in Japan. All hospitalized patients who were administered bevacizumab between January 1, 2016 and June 30, 2019 were included. |
| Participants | 6 | (*a*) *Cohort study*—Give the eligibility criteria, and the sources and methods of selection of participants. Describe methods of follow-up  *Case-control study*—Give the eligibility criteria, and the sources and methods of case ascertainment and control selection. Give the rationale for the choice of cases and controls  *Cross-sectional study*—Give the eligibility criteria, and the sources and methods of selection of participants | 5 | The index day (day 1) was defined as the first day of bevacizumab administration within the inclusion period. The exclusion criteria were as follows: receipt of bevacizumab within six months prior to day 1; age <20 years; an outlier value for weight (<25 kg) or height (<100 cm); proteinuria on or before day 1; lack of laboratory test results pertaining to the period after day 1; and receipt of dialysis within 12 months after day 1. |
|  |  | (*b*) *Cohort study*—For matched studies, give matching criteria and number of exposed and unexposed  *Case-control study*—For matched studies, give matching criteria and the number of controls per case |  | No applicable. |
| Variables | 7 | Clearly define all outcomes, exposures, predictors, potential confounders, and effect modifiers. Give diagnostic criteria, if applicable | 6, 7, Fig. 1 | The outcome of this study was an event of proteinuria within 12 months of the index day. Proteinuria positivity was defined as qualitative proteinuria ≥1+, ≥10 mg/dL spot urine protein, or ≥120 mg/day protein in 24-hour urine collection samples. The proteinuria grade was determined based on the result of the first positive qualitative value according to the Common Terminology Criteria for Adverse Events (CTCAE) v5.0: grade 1: 1+; grade 2: 2-3+, grade 3: 4+.  The antihypertensive agent used on the index day was defined by the presence of one of the following [14] (Fig 1): 1) the medication was prescribed on the index day, 2) the days supplied by the previous prescription covered the index day, or 3) the sum of the days supplied between day -27 and day 0 was more than 28. |
| Data sources/ measurement | 8* | For each variable of interest, give sources of data and details of methods of assessment (measurement). Describe comparability of assessment methods if there is more than one group | 5-8 | The NHO covers more than eight million patients in 140 hospitals throughout Japan. It provides two databases: the NHO Clinical Data Archives (NCDA), which collects real-time clinical information from the electronic medical records of NHO hospitals, and the Medical Information Analysis (MIA) data bank, which collects daily insurance claims information [12]. Sixty-six NHO hospitals and all NHO hospitals record NCDA and MIA data. Vital signs and laboratory data were obtained from the NCDA. The MIA includes data for the Diagnosis Procedure Combination/Per-Diem Payment System. The database includes patient information on demographics, primary diagnosis, comorbidities present on admission, complications occurring during hospitalization, medical procedures, medications, and materials; it also contains patient details including age, sex, body height, weight, grade of activities of daily life on admission, discharge converted to the Barthel index [13], nursing dependency score, and discharge status. International Statistical Classification of Diseases and Related Health Problems, 10th Revision (ICD-10), codes (S1 Table) were used to group cancers.  Patient characteristics included the following (S2 Table): age, sex, weight, height, body mass index (BMI), nursing dependency score associated with patient performance status [15] on the index day, Charlson Comorbidity Index (CCI) excluding cancer [16,17], other comorbidities (congestive heart failure, diabetes, or kidney disease), cancer type based on all diagnoses, previous chemotherapy use, death, chemotherapy (L01A, L01B, L01C, L01D, L01E, L01X, and L02B in the Anatomical Therapeutic Chemical Classification by World Health Organization), and hypertensive treatment on the index day (ARB, ACEI, calcium channel blocker, loop diuretics, thiazide, beta-blockers, spironolactone, and nitrates). The following vital signs and laboratory data measured on the index day or one day prior were used as variables: systolic blood pressure (SBP), diastolic blood pressure (DBP), serum creatinine (Scr), estimated glomerular filtration rate (eGFR), creatinine clearance (Ccr), albumin, blood urea nitrogen, total bilirubin, and glycated hemoglobin (HbA1c). eGFR and Ccr were calculated using the Scr value and categorized based on the Evidence-Based Practice Guideline for the Treatment of Chronic Kidney Disease 2018 released by the Japanese Society of Nephrology. |
| Bias | 9 | Describe any efforts to address potential sources of bias | 5 | All hospitalized patients who were administered bevacizumab between January 1, 2016 and June 30, 2019 were included. |
| Study size | 10 | Explain how the study size was arrived at | 5 | All hospitalized patients who were administered bevacizumab between January 1, 2016 and June 30, 2019 were included. The index day (day 1) was defined as the first day of bevacizumab administration within the inclusion period. The exclusion criteria were as follows: receipt of bevacizumab within six months prior to day 1; age <20 years; an outlier value for weight (<25 kg) or height (<100 cm); proteinuria on or before day 1; lack of laboratory test results pertaining to the period after day 1; and receipt of dialysis within 12 months after day 1. |

Continued on next page

| Quantitative variables | 11 | Explain how quantitative variables were handled in the analyses. If applicable, describe which groupings were chosen and why | 6 | Proteinuria positivity was defined as qualitative proteinuria ≥1+, ≥10 mg/dL spot urine protein, or ≥120 mg/day protein in 24-hour urine collection samples. |
| --- | --- | --- | --- | --- |
| Statistical methods | 12 | (*a*) Describe all statistical methods, including those used to control for confounding | 8, 9 | Patient characteristics are described using the mean and standard deviation for continuous variables and proportions for categorical variables. Predictor variables were compared between patients with and without proteinuria using the chi-square test for categorical variables and Wilcoxon rank-sum test for continuous variables. Multivariate logistic regression analysis was performed to determine the factors associated with the occurrence of proteinuria using the forced entry method. Variables with a P <0.15 were determined using univariate analysis, and antihypertensive drugs were selected for this analysis. Additionally, we compared chemotherapy and antihypertensive drug utilization between patients with and without proteinuria using the chi-square test. Statistical analyses were performed using JMP Pro 15 (SAS Institute Inc., Cary, North Carolina, USA), and P-values less than 0.05 indicated significant differences. |
|  |  | (*b*) Describe any methods used to examine subgroups and interactions |  | No applicable. |
|  |  | (*c*) Explain how missing data were addressed | 8 | If the Scr value was missing, it was calculated by the Modification of Diet in Renal Disease equation for Japanese patients [18], assuming a GFR of 75 mL/min/1.73 m^2^ [19]. |
|  |  | (*d*) *Cohort study*—If applicable, explain how loss to follow-up was addressed  *Case-control study*—If applicable, explain how matching of cases and controls was addressed  *Cross-sectional study*—If applicable, describe analytical methods taking account of sampling strategy |  | No applicable. |
|  |  | (*e*) Describe any sensitivity analyses |  | No applicable. |
| Results | | | | |
| Participants | 13* | (a) Report numbers of individuals at each stage of study—eg numbers potentially eligible, examined for eligibility, confirmed eligible, included in the study, completing follow-up, and analysed | 9 | Fig 2 shows the flow of the participant selection process. In total, 3,134 patients were administered bevacizumab during the study period. After exclusions, 2,458 patients were eligible for analysis. Of these patients, 654 (26.6%) experienced an episode of proteinuria within 12 months after the index day, and 1,804 (73.4%) did not experience such an episode. After the exclusion of 386 patients who were diagnosed based on a 24-hour urine collection or spot urine quantitative test, 316 (81.9%) and 70 (18.1%) patients exhibited grade 1 and grade 2 proteinuria, respectively. None of the patients exhibited grade 3 proteinuria. |
|  |  | (b) Give reasons for non-participation at each stage | Figure 2 | - Received bevacizumab within 6 months prior to index day: 510 patients - Outlier value for age, weight, or height: 13 patients - Lack of laboratory test data after index day: 70 patients - Proteinuria positive on the day before index date or on the index date: 79 patients - Dialysis during the observation period: 6 patients |
|  |  | (c) Consider use of a flow diagram | Figure 2 | Figure 2 |
| Descriptive data | 14* | (a) Give characteristics of study participants (eg demographic, clinical, social) and information on exposures and potential confounders | Table 1, 2 | Table 1, 2 |
|  |  | (b) Indicate number of participants with missing data for each variable of interest | Table 1, 2 | Table 1, 2 |
|  |  | (c) *Cohort study*—Summarise follow-up time (eg, average and total amount) |  | No applicable. |
| Outcome data | 15* | *Cohort study*—Report numbers of outcome events or summary measures over time | Figure 2, Table 1, 2, 3, 4 | Figure 2, Table 1, 2, 3, 4 |
|  |  | *Case-control study—*Report numbers in each exposure category, or summary measures of exposure |  | No applicable. |
|  |  | *Cross-sectional study—*Report numbers of outcome events or summary measures |  | No applicable. |
| Main results | 16 | (*a*) Give unadjusted estimates and, if applicable, confounder-adjusted estimates and their precision (eg, 95% confidence interval). Make clear which confounders were adjusted for and why they were included | 17 | The results of the multivariable analysis conducted to detect the factors associated with proteinuria after bevacizumab administration are presented in Table 3. Ovarian cancer was significantly associated with proteinuria (reference, colorectal cancer; odds ratio (OR), 1.6347; 95% confidence interval (CI), 1.1909–2.2440; P = 0.0024). Patients who exhibited a nursing dependency score ≥1 had greater odds of proteinuria than those with a nursing dependency score of 0 (OR, 2.3984; 95% CI, 1.8878–3.0473; P <0.0001). Patients with SBP ≥140 mmHg had higher odds of proteinuria than those with SBP <140 mmHg (OR, 1.4547; 95% CI, 1.1754–1.8002; P = 0.0006). Moreover, patients with an eGFR 60–89, 45–59, and <45 mL/min/1.73 m2 had higher odds of proteinuria than those with an eGFR ≥90 mL/min/1.73 m2 (OR, 1.3195 [95% CI, 0.9490–1.8347], P = 0.0992; OR, 1.8242 [95% CI, 1.1211–2.9683], P = 0.0155; and OR, 1.7083 [95% CI, 0.8106–3.600], P = 0.1592, respectively). Those who received RAA inhibitors on the index day had lower odds of proteinuria than patients with did not take any antihypertensives, but not significantly so (OR, 0.7285; 95% CI, 0.5071–1.0466; P = 0.0866). |
|  |  | (*b*) Report category boundaries when continuous variables were categorized | Table 1, 2, 3, 4 | Table 1, 2, 3, 4 |
|  |  | (*c*) If relevant, consider translating estimates of relative risk into absolute risk for a meaningful time period |  | No applicable. |

Continued on next page

| Other analyses | 17 | Report other analyses done—eg analyses of subgroups and interactions, and sensitivity analyses |  | No applicable. |
| --- | --- | --- | --- | --- |
| Discussion | | | | |
| Key results | 18 | Summarise key results with reference to study objectives | 23-27 | In the present study, we investigated the incidence and risk factors of bevacizumab-related proteinuria and examined the effectiveness of antihypertensive drugs in preventing proteinuria. Our results showed that approximately 27% of the patients developed proteinuria and that the factors associated with proteinuria included nursing dependence, SBP ≥140 mmHg, and low eGFR. Additionally, ovarian cancer was a risk factor for proteinuria. No significant relationship was observed between antihypertensive drugs and the occurrence of proteinuria.  In our real-world study, the proportion of patients with cancer who developed proteinuria after bevacizumab administration was higher than that reported in the package insert and a previous meta-analysis [5]. Although we excluded patients with a history of bevacizumab administration, the incidence rate was >2-fold higher than that reported in the meta-analysis. This suggests the need to closely monitor patients for proteinuria after the initial administration of bevacizumab.  Patients with ovarian cancer had significantly higher odds of exhibiting proteinuria than those with colorectal cancer. A previous study suggested that ovarian cancer is a risk factor because the bevacizumab dose used for ovarian cancer (15 mg/kg) is higher than that used for colorectal cancer (5 or 7.5 mg/kg) [7]. Our results showed no correlation between the initial dose of bevacizumab and proteinuria in the bivariate analysis; however, the dose of bevacizumab during the observation period was not surveyed in this study. Therefore, it is necessary to determine the relationship between the cumulative dose of bevacizumab and proteinuria.  In patients who exhibited nursing dependence, the odds of exhibiting proteinuria were 2.4-fold higher than those in patients who were nursing independent. There was no association between proteinuria and age, sex, BMI, CCI, or other comorbidities. We hypothesized that the presence of comorbidities, especially cardiovascular and kidney diseases, would be risk factors because the pathology of proteinuria is associated with hypertension [20]. In this study, kidney function at the administration of bevacizumab was significantly worse in patients with proteinuria than in those without proteinuria. This finding suggests that it is important to monitor kidney function to prevent or manage proteinuria when bevacizumab is used.  Patients with proteinuria had significantly higher SBP and DBP on the index day than those without proteinuria, and SBP ≥140 mmHg was associated with an OR of 1.45 compared with SBP <140 mmHg in the multivariable analysis. According to a previous study, bevacizumab-associated hypertension is likely to occur in patients with pre-existing hypertension [21]. Because hypertension and proteinuria are related, blood pressure should be monitored from the start of bevacizumab therapy. A lower eGFR than ≥90 mL/min/1.73 m^2^ was associated with higher odds of proteinuria; however, the association of proteinuria with an eGFR <45 mL/min/1.73 m^2^ was not significant. We did not have a sufficient number of patients in this group (with low eGFR) because we excluded patients who underwent dialysis, and bevacizumab may not have been selected for patients with renal dysfunction. This could explain the lack of significance of these results.  Because bevacizumab is administered in combination with other anticancer agents, we investigated the type of chemotherapeutic administered on the index day. Platinum-based and antimetabolite-based drugs were administered along with bevacizumab in more than half of patients. As platinum-based chemotherapy causes renal toxicity [22], we expected such therapy to influence proteinuria. However, the risk of proteinuria was not significantly higher in patients who used platinum-based agents. When platinum-based agents are used, hydration treatment is administered to prevent renal toxicity, and healthcare professionals monitor renal function [23]. Our findings suggest that patients who received platinum-based agents with bevacizumab could have been monitored adequately so that proteinuria was eventually avoided. In contrast, the proportion of patients who received plant alkaloid-based agents on the index day was significantly higher among those with proteinuria than among those without proteinuria. Taxanes are representative plant alkaloid-based drugs administered to patients with female-specific cancers such as ovarian, breast, and cervical cancer. However, the administration of bevacizumab in combination with taxanes does not influence the toxicity profile of taxanes [24,25]. In our study, although we did not include sex in the multivariable analysis, we adjusted for the type of cancer. Therefore, patients with ovarian cancer treated with plant alkaloid-based drugs could be at risk of developing proteinuria caused by bevacizumab, although further studies are required to clarify this.  To evaluate the effectiveness of antihypertensive drugs in preventing proteinuria, we focused on their use on the index day. Although approximately 30% of patients had hypertension based on the laboratory data on the index day, approximately 80% of patients who received bevacizumab did not take any antihypertensive drugs. Among the patients who received antihypertensive drugs, approximately 65% were treated with RAA inhibitors alone or in combination with other types of antihypertensive drugs. The type of antihypertensive drug administered on the index day was not associated with proteinuria. We hypothesized that RAA inhibitors could prevent proteinuria caused by bevacizumab. However, we found no association between RAA inhibitor use on the index day and proteinuria. A previous study on the use of antihypertensives during bevacizumab therapy reported that higher doses of a single agent or more than one antihypertensive agent are required to normalize blood pressure [21]. This implies that the duration of antihypertensive use should be investigated in further studies to reveal their effect on proteinuria. |
| Limitations | 19 | Discuss limitations of the study, taking into account sources of potential bias or imprecision. Discuss both direction and magnitude of any potential bias | 27, 28 | The present study had some limitations. First, we did not investigate the duration of bevacizumab and antihypertensive drug use after the index day. A previous long-term extension study of bevacizumab administration showed that adverse effects occur in 56.8% of patients with cancer, the most common of which were proteinuria (41.1%) and hypertension (10.5%) [26]. Thus, continuous use of these drugs may affect outcomes. Second, the observation period was only 12 months. Data on the incidence of proteinuria after 12 months are required to understand the long-term adverse effects. Finally, because we used clinical data, it is possible that treatment such as hydration therapy administered in conjunction with platinum-based chemotherapy ameliorates the risk of proteinuria, which may affect our results. |
| Interpretation | 20 | Give a cautious overall interpretation of results considering objectives, limitations, multiplicity of analyses, results from similar studies, and other relevant evidence |  | In conclusion, we found that the incidence of proteinuria among patients who were administered bevacizumab was more than two-fold higher than that described in the package insert. We also found that nursing dependence, SBP ≥140 mmHg, and low eGFR at the time of bevacizumab administration were risk factors for proteinuria. The type of antihypertensive drug administered after bevacizumab did not significantly affect proteinuria occurrence. |
| Generalisability | 21 | Discuss the generalisability (external validity) of the study results |  | These findings suggest that the incidence of proteinuria is higher than currently reported. Therefore, patients on bevacizumab may be at a higher risk of proteinuria and should be monitored carefully. |
| Other information | |  | | |
| Funding | 22 | Give the source of funding and the role of the funders for the present study and, if applicable, for the original study on which the present article is based |  | No applicable. |

*Give information separately for cases and controls in case-control studies and, if applicable, for exposed and unexposed groups in cohort and cross-sectional studies.

**Note:** An Explanation and Elaboration article discusses each checklist item and gives methodological background and published examples of transparent reporting. The STROBE checklist is best used in conjunction with this article (freely available on the Web sites of PLoS Medicine at http://www.plosmedicine.org/, Annals of Internal Medicine at http://www.annals.org/, and Epidemiology at http://www.epidem.com/). Information on the STROBE Initiative is available at www.strobe-statement.org.
