# Supplementary material for: Bevacizumab-induced proteinuria and its association with antihypertensive drugs: A retrospective cohort study using a Japanese administrative database
Source: PLoS One. 2023 Aug 10;18(8):e0289950. doi: 10.1371/journal.pone.0289950 (PMC10414654; doi:10.1371/journal.pone.0289950)
Supplement: S1 Table — (DOCX) [file pone.0289950.s002.docx]

**S1 Table. ICD-10 codes for identification of cancer type.**

| Colorectal cancer | C182, C184, C185, C186, C187, C189, C19, D010, D011, D374, C20, C785, D012, D375 |
| --- | --- |
| Non-small cell lung cancer | C340-343, C349 |
| Ovarian cancer | C56, C796, C799 |
| Cervical cancer | C530, C538, C539, D060, D069 |
| Breast cancer | C500-506, C508, C509, C792, C795, C798 |
| Malignant glioma | C719 |
| Other | Any code starting from “C” excluding the ones listed above. |
