# Supplementary material for: Bevacizumab-induced proteinuria and its association with antihypertensive drugs: A retrospective cohort study using a Japanese administrative database
Source: PLoS One. 2023 Aug 10;18(8):e0289950. doi: 10.1371/journal.pone.0289950 (PMC10414654; doi:10.1371/journal.pone.0289950)
Supplement: S2 Table — (DOCX) [file pone.0289950.s003.docx]

**S2 Table. Variables list.**

| Nursing dependency score | Including 7 items: wound care, respiratory care, management of more than three intravenous infusions, electrocardiographic monitoring, syringe pump management, transfusion or blood product management, other professional treatment or care (e.g. narcotic drug). Patients were assigned a score of 0 or 1 (“without assistance” and “with assistance”) depending on patient condition. The sum of these seven items was used for this variable. |
| --- | --- |
| Charlson Comorbidity Index (CCI) | The CCI scores were calculated according to the Quan’s coding algorithms^12, 13)^ excluding for CCI Condition number 11 (cancer) and CCI Condition number 15 (metastatic carcinoma). |
| Congestive heart failure | ICD-10 code: I43, 50, 099, 110, 130, 132, 255, 420, 425-429, and P290 |
| Diabetes | ICD-10 code: E100-149 |
| Kidney disease | ICD-10 code: N00-39 |
| Five commonly occurring cancers | ICD-10 code: lung cancer (C34), colorectal cancer (C18), breast cancer (C50), gastric cancer (C16), and liver cancer (C22) |
