# Supplementary material for: Bevacizumab-induced proteinuria and its association with antihypertensive drugs: A retrospective cohort study using a Japanese administrative database
Source: PLoS One. 2023 Aug 10;18(8):e0289950. doi: 10.1371/journal.pone.0289950 (PMC10414654; doi:10.1371/journal.pone.0289950)
Supplement: Attachment — Submitted filename: Bevacizumab induced proteinuria.docx [file pone.0289950.s004.docx]

In this study

Age, BMI, Charlson Index, type of Cancer, previous Chemotherapy and death were not associated with protU.

Lower eGFR is associated with protU but without a direct correlation

Predictive risk factors for protU after use of bevacizumab were nursing dependency and systolic blood pressure.

It is necessary to define nursing dependency in this population; you should compare patients with and without nursing dependency. This will possibly help to clarify and identify the factors associated with development of protU in this population. The comorbidity index that could be associated with nursing dependency did not differentiate the development of protU.

Were the renin-angiotensin-aldosterone inhibitors, used to prevent proteinuria, not useful in these patients?

The Table results is difficult to read. It is presented in successive pages and the first line of new pages has no information – ProtU, noProtU and p-value should be added in every new page
