# Supplementary material for: Bevacizumab-induced proteinuria and its association with antihypertensive drugs: A retrospective cohort study using a Japanese administrative database
Source: PLoS One. 2023 Aug 10;18(8):e0289950. doi: 10.1371/journal.pone.0289950 (PMC10414654; doi:10.1371/journal.pone.0289950)
Supplement: Attachment — Submitted filename: Response to Reviewers_2.docx [file pone.0289950.s005.docx]

­TO:	Dr. Mirosława Püsküllüoğlu, MD, PhD., Academic Editor, PLOS ONE

RE:	Revision submission

DATE:	March 30, 2023

Dear Dr. Mirosława Püsküllüoğlu:

Thank you very much for your e-mail dated March 1, 2023, regarding our manuscript titled, **“Bevacizumab-induced proteinuria and its association with antihypertensive drugs: a retrospective cohort study using a Japanese administrative database,”** coauthored by Anna Kiyomi, Fukiko Koizumi, Shinobu Imai, Hayato Yamana, Hiromasa Horiguchi, Kiyohide Fushimi, and Munetoshi Sugiura. The manuscript ID is **PONE-D-23-01693.** We appreciate the comments from the reviewers, which have greatly improved the quality of our manuscript. We have provided point-by-point responses to the reviewers’ comments, as shown below, and have addressed and clarified the reviewers’ concerns.

Regarding the data availability statement, the data contain potentially identifying or sensitive patient information, and the National Hospital Organization (NHO) has imposed them. The contact information: [700-dbproject@mail.hosp.go.jp](mailto:700-dbproject@mail.hosp.go.jp); Website (Japanese only): <https://nho.hosp.go.jp/cnt1-1_000070.html>. I clarified with the NHO that anyone can get the dataset once you contact them. Additionally, we did not have any special access or request privileges that others would not have. This data was purchased from our grant. Once it is approved by the Research Ethics Committee and the Data Access Committee of the NHO, you can access the data on-site (mandatory) after the fee payment. No one can bring the data outside of their secured analysis room, even though the data is anonymous. Because of this reason, the data is legally restricted. In order to provide the information of the National Hospital Organization Database, I have attached the Databases available for pharmacoepidemiology research in Japan issued by the Japanese Society for Pharmacoepidemiology and the statement of this regulation (see page 9, 8. Data Utilization, 1. Data Utilization policy, the third bullet) as evidence.

**Response to reviewers**

Reviewer #1: In this study, Age, BMI, Charlson Index, type of Cancer, previous Chemotherapy and death were not associated with protU. Lower eGFR is associated with protU but without a direct correlation. Predictive risk factors for protU after use of bevacizumab were nursing dependency and systolic blood pressure.

It is necessary to define nursing dependency in this population; you should compare patients with and without nursing dependency. This will possibly help to clarify and identify the factors associated with development of protU in this population. The comorbidity index that could be associated with nursing dependency did not differentiate the development of protU.

**Response:**

We appreciate the reviewer for taking the time to review our manuscript and provide valuable feedback. As the reviewer mentioned below, our table was difficult to read, and we realize that it might have caused misreading regarding nursing dependency. We did indeed compare nursing dependency (with and without) among the patients with and without proteinuria (Table 1 and line 175. Moreover, patients who exhibited a nursing dependency score ≥1 had greater odds of proteinuria than those with a nursing dependency score of 0 (OR, 2.3984; 95% CI, 1.8878-3.0473; P <0.0001). This is exactly what the reviewer mentioned and is shown in Table 3. We modified the tables to make them easier to read and added the definition of nursing dependency in lines 123-125.

Were the renin-angiotensin-aldosterone inhibitors, used to prevent proteinuria, not useful in these patients?

**Response:**

Thank you for pointing this out. Because our research was a cross-sectional study, we did not conduct an evaluation of proteinuria prevention using renin-angiotensin-aldosterone inhibitors in this case. Therefore, we cannot state whether the drug benefits these patients. Further study is required to understand the effects of renin-angiotensin-aldosterone inhibitors on the prevention of proteinuria. We added this content to lines 340-344.

The Table results is difficult to read. It is presented in successive pages and the first line of new pages has no information – ProtU, noProtU and p-value, etc. should be added in every new page.

**Response:**

Thank you for your comment, and we apologize for the difficulty in reading the tables. We adjusted each table to make it easier to read in the revised manuscript, but we believe that the journal editor will modify the tables again when they work on the page layout.

Reviewer #2: Dear Authors,

I have reviewed your work titled "Bevacizumab-induced proteinuria and its association with antihypertensive drugs: a retrospective cohort study using a Japanese administrative database".

It was direct with well outlined research question and objectives.

It would be interesting to note what the relationship of the occurrence of proteinuria and renal function would be if the relationship were assessed according to current CKD GFR staging 1 to 5. The authors note that the exclusion of patients on dialysis (and thus the lower patient numbers) accounted for the relationship observed between Bevacizumab and eGFR <45. However eGFR <45 encompasses a broad group of patients from CKD 3b - CKD 5 for whom proteinuria risk profiles might be different.

The authors are however correct in pointing out that there may not be enough numbers in the group of GFR < 45 due to a form of selection bias with respect to the patient who gets the medication in the first place i.e. indication bias.

As you have rightly highlighted in the limitations, the role of continued use of antihypertensives in modifying the outcome of proteinuria is one that must be borne in mind, but your study could not unfortunately evaluate. As your data was from an administrative data set, I would suspect that data can be gathered for this information and incorporated into this study.

The higher occurrence of proteinuria reported in this study than that documented in the medications drug insert does raise some concerns and hopefully triggers further research and clarifications.

**Response:**

We appreciate the reviewer for taking the time to review our manuscript and provide valuable feedback. As the reviewer mentioned, we categorized the patients based on their renal function to conduct the analysis. Although there were some limitations in this study, we revealed real-world evidence that proteinuria occurrence in patients who were administrated bevacizumab was higher than what the clinical study showed. Likewise, as the reviewer mentioned, we believe this finding could be beneficial in clinical practice for patients administrated bevacizumab and that further studies detecting the prevention of proteinuria by the renin-angiotensin-aldosterone inhibitors should be conducted. Therefore, based on the reviewer’s comments, we feel no additional changes are needed.

Thank you again to the reviewers and editorial team for reviewing our manuscript and providing constructive feedback regarding our research. The modified portions are colored in red in the revised manuscript. We believe that the revised manuscript, which addresses the reviewer’s concerns, is now ready for publication in *PLOS ONE*.

Thank you for your time, and we look forward to hearing your decision soon.

Sincerely,

Anna Kiyomi, PhD.

Assistant Professor

Tokyo University of Pharmacy and Life Sciences

1432-1 Horinouchi, Hachioji

Tokyo 192-0392, Japan

Tel/Fax: +81-42-676-6622

[akiyomi@toyaku.ac.jp](mailto:akiyomi@toyaku.ac.jp)
