# Supplementary material for: Bevacizumab-induced proteinuria and its association with antihypertensive drugs: A retrospective cohort study using a Japanese administrative database
Source: PLoS One. 2023 Aug 10;18(8):e0289950. doi: 10.1371/journal.pone.0289950 (PMC10414654; doi:10.1371/journal.pone.0289950)
Supplement: Attachment — Submitted filename: Response_to_Reviewers_0715.docx [file pone.0289950.s006.docx]

­TO:	Dr. Mirosława Püsküllüoğlu, MD, PhD., Academic Editor, PLOS ONE

RE:	Resubmission of manuscript [PONE-D-23-01693]

DATE:	July 15, 2023

Dear Dr. Mirosława Püsküllüoğlu:

Thank you for your e-mail dated June 5, 2023, regarding our manuscript titled, **“Bevacizumab-induced proteinuria and its association with antihypertensive drugs: A retrospective cohort study using a Japanese administrative database,”** coauthored by Anna Kiyomi, Fukiko Koizumi, Shinobu Imai, Hayato Yamana, Hiromasa Horiguchi, Kiyohide Fushimi, and Munetoshi Sugiura. The manuscript ID is **PONE-D-23-01693.**

We appreciate the comments from the reviewers, which have greatly improved the quality of our manuscript. The manuscript has been rechecked and the necessary changes have been made in accordance with the reviewers’ suggestions. We have clarified all the concerns of the reviewers and have provided point-by-point responses to all the comments, as shown below.

**Response to reviewers**

Reviewer #3:

Major remark: The core findings of the paper rely on subgroup comparisons and univariate analysis. Considering that proteinuria can be influenced by various factors, such as hypertension, diabetes, and obesity (Ong et al., Nephrology (Carlton), 2013), it is advisable to standardize the presented data to account for these factors and eliminate their influence on the established results. I recommend consulting with a statistician and considering the incorporation of multivariate analysis with standardization to the aforementioned factors.

**Response:**

We appreciate the reviewer for taking the time to review our manuscript and for providing valuable feedback. As the reviewer mentioned, several factors, particularly obesity and diabetes, could have effects on proteinuria. As we already used the variables of blood pressure, we have added obesity (BMI) and diabetes to the multivariate logistic regression analysis. The methods and results are presented in lines 153, 218−232, and Table 3. Following the additional analyses, our results did not change.

Minor remarks:

Line 82: It would be beneficial to provide an explanation for why weight and height values were considered exclusion criteria.

**Response:**

Thank you for your suggestion. We have excluded the patients with weight of <25 kg or height of <100 cm; they are considered to have been registered incorrectly, with values such as 0 or to have switched weight and height values. We have added the information in lines 82−84.

Line 109-110: Could you please clarify whether the proteinuria values mentioned are equal? If so, providing a reference for this information would be helpful.

**Response:** Thank you for your suggestion. Although these values are not necessarily the same, the Common Terminology Criteria for Adverse Events (CTCAE) v5.0 cites the definition and proteinuria positivity values. We have inserted this information in lines 112−113.

Line 129: It would be beneficial to specify the chemotherapeutic agents coded in the Anatomical Therapeutic Chemical (ATC) classification. Are they the same as those listed in Table 4?

**Response:** Thank you for your suggestion. As the reviewer mentioned, the chemotherapeutic agents coded in the ATC classification are the same as those listed in Table 4. Therefore, we have added this content in lines 130−132 to specify the codes; L01A: alkylating-based, L01B: antimetabolites-based, L01C: plant alkaloid-based, L01D: cytotoxic antibiotics-based, L01E: protein kinase inhibitor-based, L01X: platinum-based, and L02B: hormone antagonist-based agents.

Line 164-165: The information provided here is somewhat unclear and may not be necessary. If you intended to convey that during observation period, 15.1% of patients with proteinuria and 15.2% of patients without proteinuria died, it is important to provide more precise details or at least specify the time period during which these deaths occurred and the reasons behind them. Otherwise, readers may mistakenly assume that proteinuria was the direct cause of death in these individuals.

**Response:** Thank you for your valuable suggestion. As the reviewer suggested, because this study was conducted as a cross-sectional study, it is impossible to discuss further about why and how the death occurred. Consequently, we have removed the content from the revised manuscript.

Table 1: Is it possible to include p-values for the different types of cancers rather than providing only general information for all diseases? I would suggest replacing the +/- symbols in the "chemotherapy before the index day" row with descriptive terms such as "Applied" and "Non-applied." Additionally, since readers may not be familiar with the International Classification of Diseases (ICD) codes, it would be helpful to provide their specific definitions in a footnote.

**Response:** Considering the reviewer’s comment, we have added each p-value for the different types of cancers and modified the results (lines 182–184). The symbols regarding chemotherapy in Table 1 were changed to “Applied” and “Non-applied.” We have also included the definitions of the ICD code in a footnote.

Regarding lines 212-225, as detailed data are already presented in Table 3, I recommend avoiding repetition and transforming this paragraph into a more narrative and descriptive form. For example, you could state that XYZ increases the risk of proteinuria by 20%.

**Response:**

Thank you for your suggestion. According to the reviewer’s recommendations, we have modified the explanation to make them easier to understand in lines 219–232.

Thank you once again to the reviewers and editorial team for reviewing our manuscript and providing constructive feedback regarding our research. The modified portions are colored in red in the revised manuscript. We believe that the revised manuscript addresses the reviewers’ concerns and is now ready for publication in *PLOS ONE*.

Thank you for your time, and we look forward to hearing your decision soon.

Sincerely,

Anna Kiyomi, PhD.

Assistant Professor

Tokyo University of Pharmacy and Life Sciences

1432-1 Horinouchi, Hachioji

Tokyo 192-0392, Japan

Tel/Fax: +81-42-676-6622

akiyomi@toyaku.ac.jp
